# Origins of cellular geometry

**DOI:** 10.1186/1741-7007-9-57

**Published:** 2011-08-31

**Authors:** Wallace F Marshall

**Affiliations:** 1 Department of Biochemistry and Biophysics, University of California, San Francisco, CA 94143-2200, USA

## Abstract

Cells are highly complex and orderly machines, with defined shapes and a startling variety of internal organizations. Complex geometry is a feature of both free-living unicellular organisms and cells inside multicellular animals. Where does the geometry of a cell come from? Many of the same questions that arise in developmental biology can also be asked of cells, but in most cases we do not know the answers. How much of cellular organization is dictated by global cell polarity cues as opposed to local interactions between cellular components? Does cellular structure persist across cell generations? What is the relationship between cell geometry and tissue organization? What ensures that intracellular structures are scaled to the overall size of the cell? Cell biology is only now beginning to come to grips with these questions.

## 

The complex structure of the living cell is critical for cellular function. Indeed, it has recently been argued that the spatial organization of the cell is even more important for cellular properties than is its genetic, epigenetic, or physiological state [1]. Yet relatively little is known about the mechanisms that produce the complex spatial organization of a living cell. Understanding the mechanisms that generate pattern and organization in cells has been identified as a key challenge for the new millennium [2,3]. Here I consider the extent of cellular complexity in both free-living cells and cells in metazoan tissues, and ask whether any general organizational principles can be identified.

## Complex structures inside single cells

The dramatic advances in the understanding of molecular and biochemical processes over the last half century or so have understandably shifted the focus of cell biology from the structural features of cells in which it had its beginnings. Nevertheless, it has long been recognized that cells show a high degree of reproducible, non-random geometrical order, the most striking being the elaborate structural specializations of some free-living single-celled organisms.

**Figure 1:**
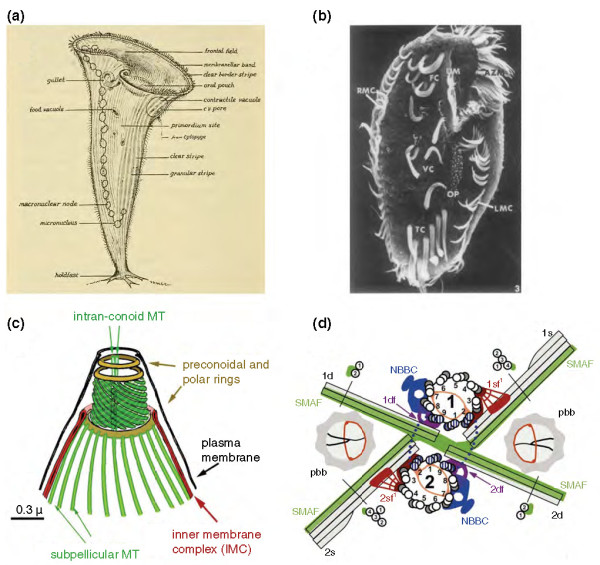
**Complexity in free-living eukaryotic cells**. **(a) **The giant ciliate *Stentor coeruleus*, a classic system for studying cellular pattern formation using microsurgical methods [5]. Each cell can be up to 2 mm long and has a complex and highly asymmetrical morphology that can be faithfully regenerated following surgical manipulation. Image courtesy of Biodiversity Heritage Library. http://www.biodiversitylibrary.org[5]. **(b) **Ventral surface of *Stylonychia *[7] showing distinct classes of cirri arranged in highly asymmetrical patterns that are reproducible from cell to cell. Reprinted from *Developmental Biology *[7] with permission from Elsevier. **(c) **Apical complex (from which the apicomplexans take their name) of *Toxoplasma *cell [9] containing distinct sets of microtubule-based structures. **(d) **Basal apparatus of *Chlamydomonas *[11] showing the complex inter-relationship between the two mature basal bodies, the two daughter basal bodies formed prior to division, four microtubule-based rootlets, and several accessory fibers linking the rootlets to the basal bodies. These complex geometrical relations surrounding centrioles and basal bodies are likely a key source of local positional information. Reproduced with permission from *Journal of Cell Science *[11].

Many of the most complex-looking cells are free-living protists, especially the ciliates [4], which can contain tens of thousands of cilia organized into rows and whorls. One of the most remarkable of these is *Stentor coeruleus *(Figure 1a), a millimeter-long cell that has a clearly recognizable anterior-posterior axis, with a mouth at one end and a holdfast structure at the other. The ciliary rows, which run along the anterior-posterior axis, have a variable spacing between successive rows such that rows become increasingly close together as they run counter-clockwise around the equator of the animal. Thus the cell also shows an inherent chirality and left-right asymmetry. The ventral region of the cell, where the most closely spaced rows meet the most widely spaced rows, defines the position where a new mouthpart forms during cell division. If the pre-existing mouth is severed using microsurgery, the cell can grow a new mouth whose formation begins with a primordium that develops at the same site on the ventral surface. Moreover, the same region, if transplanted to another cell using microsurgery, is capable of inducing formation of an ectopic mouth [6]. Thus the ventral region of this single cell behaves in a manner analogous to that of organizer regions in the development of metazoa. It thus appears that a single cell can manifest all of the hallmarks of animal developmental biology: axiation, left-right asymmetry, pattern formation, organizers, and regeneration.

The complexity of cortical patterning is even more striking in hypotrichous ciliates such as *Stylonychia *(Figure 1b), whose ventral surface contains an asymmetrical set of distinct cilia-based structures called cirri, formed by groups of cilia fused together. These cirri occur in highly reproducible patterns, with each cirrus found in a reproducible position relative to the anterior-posterior and left-right axes [7], and have provided the basis for experiments on the relative importance of local and global positional cues for pattern formation, discussed later in this article.

Many other free-living protists can form extraordinarily elaborate ordered structures with diverse specialized functions. Apicomplexan parasites, which include the *Plasmodium *species that cause malaria, are named for the apical complex - an exceedingly regular and complex set of microtubule-based structures at their apical end (Figure 1c) that somehow acts as a machine to drive cellular invasion [8,9]. As another example, some dinoflagellates form an array of lipid droplets into a reflective lens that focuses light onto a patch of photoreceptors located in the base of their flagella [10].

The invasive machinery of the apicomplexans and the eyespots of the dinoflagellates are very specialized structures. A more general building block for complex structures in cells is the centriole, a nine-fold symmetric barrel of microtubule triplets. The most dramatic examples of complex structures built from centrioles are the cortical arrays of the ciliates (Figure 1b), which consist of linear arrays of hundreds of centrioles linked together, each of which acts as a basal body to nucleate a microtubule-based motile cilium. Although the centriole arrays in ciliates are a particularly extreme example, in fact most free-living cells have complex structures associated with their basal bodies in highly defined geometries - for example, *Chlamydomonas*, a unicellular green alga related to the evolutionary ancestors of land plants, has a set of four microtubule-based rootlets attached to the centrioles by a set of proteinaceous fibers (Figure 1d). These rootlets in turn determine the position of other structures in the cell [12].

**Figure 2:**
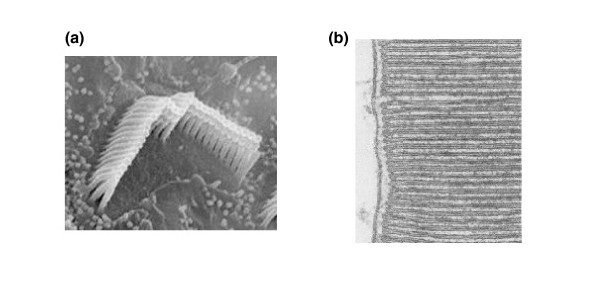
**Complex intracellular structures in animal cells**. **(a) **Stereocilia bundles [13]. **(b) **Retinal rod outer segment [14] showing well-ordered stacks of rhodopsin-containing membrane vesicles. Reproduced with permission from *Journal of Neuroscience *[66].

Cellular structure can be just as complex in the cells of multicellular organisms as it can in unicellular ones: the two examples in Figure 2 illustrate the very complex and distinct structures that can form in different metazoan cells in a single organism. To what extent are the obvious and sometimes spectacular morphological specializations of some unicellular organisms and specialized vertebrate cell types a reflection of a universal property of cells? Most mammalian cells in culture look more or less like amorphous blobs. Do such blob-like cells actually have a shape?

In one approach to testing for defined shapes, Pincus and Theriot [15] and Keren *et al*. [16] devised a method for defining a space of all possible blob-like shapes and concluded that real cells explore only a nonrandom subset of this space. An alternative test is to compare sister cells and ask if their shapes are more similar than those of non-sisters, a method that yielded a positive result for some cell types [17], suggesting that some determinant of shape is transmitted from the mother cell to her daughters. Further evidence for inheritance of a large-scale spatial patterning comes from reports that sister cells tend to be mirror images of each other after division [18-21]. Mirror symmetry has long been known at the level of chromosome arrangement in the nucleus [22,23] but the degree to which other levels of cellular structure show this type of symmetry, and how long it may persist after division, remains to be explored. Some of the observations cited above suggest that determinants of cell shape can persist over generations of cells, and raise the question of how far structure seen in a cell has to be generated *de novo *in every generation, and how far it is inherited from previous generations. The answer to this question would affect how we think about cell shape determination. If shapes could persist over multiple generations, this would be a source of cell-to-cell variation comparable to epigenetic changes in gene expression, and would have important consequences for development and evolution. This question has been approached by the experiments described above, but remains to be systematically explored. It is also not yet clear precisely what the physical basis of shape transmission from mother to daughter might be. In HeLa cells, the pattern of extracellular matrix deposited by the mother cell prior to mitosis serves as a substrate for the daughter cells after cytokinesis, which thus tend each to occupy half of the mother's overall footprint [24]. Such inheritance of extracellular matrix (ECM) patterning from mother to daughters means that mother shape has the potential to affect daughter shape strongly. Although these studies were performed with cells grown on glass coverslips, cells in tissues also secrete ECM and so similar effects can be expected even in more natural contexts.

## From cells to tissues

We often draw a distinction between cell morphology and tissue morphology, but this may be a false dichotomy. One of the most provocative experiments in the history of biology, the mechanism of whose outcome remains completely unexplored, is the study by Fankhauser [25] on cells in the pronephric duct of polyploid salamanders. He found that as ploidy increased, cell size increased without any increase in the diameter of the duct, so that the number of cells seen in a cross-section dropped from five to eight in haploids to three to five in diploids, and went down to one to three in pentaploids. In pentaploids, even though there was just a single cell, that one cell folded over to create a duct lumen within itself [25]. This argues that the shape of a single cell can be greatly altered in order to produce a specified form for the overall tissue, as though the tissue were targeted to adopt a specific structure whether divided into multiple cells or not.

An extraordinary example of an analogous phenomenon was reported more than 100 years ago by Lillie [26], who treated eggs of marine polychaete worms with organic solvents to prevent cytokinesis. These embryos normally form a free-swimming trochophore larva, characterized by a bilobed appearance and tufts of cilia in defined positions. When cytokinesis was blocked mitosis continued, resulting in a syncytium. Amazingly, the massive single syncytial cell still took on an asymmetric bilobed appearance, tufts of cilia still formed, and intracellular granules partitioned, resulting in a single cell that appeared remarkably like a normal trochophore larva [26].

These results provide a tantalizing hint that there is a fundamental tendency for a tissue to form a particular overall structure, and that the same structure will tend to form regardless of how its living material is partitioned into cells. The demonstration that experimental perturbation of tissues can cause cell morphology to change so as to maintain developmental patterns of the overall tissue is powerful evidence that cell shape ultimately arises from the external environment as well as processes intrinsic to the cell. Experiments in which cells are grown on micropatterned substrates provide further evidence for the effects of external factors. When cells are forced to adhere to patterns of different shapes, there is a clear influence on cell shape [27] that is then propagated to cell behavior and internal organization [28]. In a multicellular tissue - for example, in epithelial sheets - geometrical constraints on cell shape can result from interactions with neighboring cells [29-31] as well as from the pattern of cell division [32]. Cells can also sculpt their own shapes by attaching parts of themselves to stationary structures and then migrating away [33]. Clearly the repertoire of geometry-determining mechanisms available to cells in a complex tissue could be vastly larger than that available to free living single cells.

It is also to be noted that while many aspects of cell shape and polarity may be able to self-organize through spontaneous symmetry breaking, this does not by any means preclude the possibility of external cues determining the direction in which symmetry is broken. In physics, the classic example of self-organization biased by an external cue is ferromagnetism, which has been proposed as an analogy for understanding biological organization [34]; but even in this case, an externally applied magnetic field is able to bias the direction in which a ferromagnetic material will magnetize.

Clearly, cells can have very complex and specific shapes, and these shapes are determined in response to both intrinsic and extrinsic determinants. But what are the mechanisms that actually produce shape?

## Inheritance versus self-organization

In considering the origins of cell morphology, we can delineate two extremes. On the one hand, the geometry of a cell may be entirely determined by the geometry of its parent cell, and then simply inherited. At the other extreme, each cell when born may self-organize its geometry without reference to preceding cells or external influences. Finally, the shape of a cell may be dictated entirely by the external environment, such as the positions of neighboring cells or developmental signals. The studies on cell shape in epithelial sheets discussed above support the importance of this latter influence, and one possible advantage of working with single-celled organisms is that this influence is largely absent, vastly simplifying the investigation of cell shape determination.

There is clear-cut evidence that cell shape can, to some extent, be transmitted from a mother cell to her daughters. Beisson and Sonneborn [35] demonstrated inheritance of cellular pattern most conclusively through their experiments in *Paramecium*, in which inverted ciliary rows were created and then found to propagate faithfully to progeny cells independently of any genetic change. In this case, the propagation of the inverted row orientation occurs because new basal bodies are always assembled at a precise angular location relative to pre-existing basal bodies, so that if the whole row of basal bodies is inverted, it will elongate by addition of new basal bodies in the same, incorrect, orientation. Then, when the row is partitioned during cytokinesis, both daughters inherit half of the original inverted row, which, therefore, remains inverted in both daughters. A similar template-based mode of inheritance of altered structures was demonstrated by Jennings [36] in the amoeba *Difflugia corona*, which builds a hard shell of silica particles with a single opening from which pseudopods extend. This opening is surrounded by a number of pointed projections called teeth, and Jennings showed that if some of these teeth are experimentally removed with a glass needle, the cell forms a daughter cell with a similarly reduced number of teeth. In this case, the inheritance arises because daughter cells grow their new teeth in the gaps between the teeth in the mother, so that the number of teeth tends to be similar to that of the mother. Such experiments show that extreme alterations of structure can be inherited, but how much does this type of structural inheritance contribute to the generation of normal cell morphology?

Mutations have been identified in many cases that alter cell morphology, indicating that there must be a genetic input to maintaining shape; but one could argue that these mutations may affect the maintenance of accurate copying of the morphology rather than an active role of the mutant genes in generating the morphology in the first place. Another way to test for strict inheritance is to look for spontaneous variations in cell morphology and then ask whether the altered shape is strictly maintained in lineages derived from the abnormal cell. A series of elegant experiments in ciliates identified cells with unusually large or small numbers of ciliary rows, and then followed the progeny of such cells for many generations [37,38]. The result is that the number of ciliary rows correlates strongly with the number of rows in the parent, but over a time scale of a hundred generations the average number of rows gradually returns to that seen in the general population, even starting from parents with extremely large or small numbers. Similar results were seen in studies of centriole copy number variation from cell to cell [39]. Such results argue that inheritance and cell-intrinsic processes combine to determine cell morphology.

The most decisive evidence against a purely inherited mode of morphogenesis comes from microsurgical experiments in ciliates, in which cell morphology can be drastically altered. While in the Beisson and Sonneborn experiment certain types of altered morphologies are stably inherited, experiments in *Stentor *have shown that the vast majority of structural alterations are rapidly corrected, resulting in a normal looking cell [5,6,40].

The ability of cells to correct their structures suggests they may have active mechanisms for sensing and correcting structural abnormalities. One clear example is the transcriptional response to flagellar detachment in *Chlamydomonas *[41], in which removal of the flagellum causes upregulation of hundreds of genes, most of which encode components of the flagellum [42]. The molecular pathway by which the cell senses the loss of its flagellum remains unknown.

## Local versus global information

There are two possible sources of information for directing the assembly of a cellular structure: global information about the overall polarity of the cell, and local information about the disposition of neighboring pre-existing cellular structures. Global information could be provided by several sources. First, there may be diffusible morphogens that provide long-range positional information. This seems to be the case in the *Drosophila *embryo, for example. Second, cell polarity systems involving networks of interacting proteins can set up long-range informational cues, although the mechanism by which these systems break symmetry and convey positional information is still extremely controversial. Finally, the geometric shapes of cells can directly influence the position of internal structures: for example, several studies have shown that cell geometry can directly dictate orientation of mitotic spindles [43,44]. In contrast, local information is most likely to arise from interactions between neighboring structures, presumably mediated by protein-protein interactions on the surface of the structures in question.

In principle, global information could be sufficient to define structures if it conveyed small enough differences in position. It is unclear, however, if global positional cues within a cell could have high enough resolution to specify cellular fine structure in detail. The spatial resolution of gradients based on diffusible molecules has fundamental limitations set by the ability of receptors to discriminate small differences in ligand concentration, such that sensitivity to concentration changes in one part of the gradient comes at the cost of saturation in the rest of the gradient [45]. Such considerations lead to the idea that global positional information might provide at most a low-resolution map of position within the cell that must then be refined by local determination of structure and organization.

One way to dissect the contributions of local and global information is to examine mutants that disrupt global structure and ask how local ordering of substructures is affected. For example, ciliates such as *Paramecium *are covered with parallel linear rows of cilia nucleated by corresponding rows of basal bodies. In addition to the basal bodies and cilia, these rows contain numerous other ultrastructural features, such as fibrous bundles that run alongside the rows of basal bodies, and exocytic organelles called trichocysts. Each of these structures is located in a characteristic position relative to the others, so that the ciliary row can be viewed as a repeating series of cortical units each consisting of basal bodies, trichocysts, and fibers. In the *kin241 *and *disA1 *mutants of *Paramecium *and *Tetrahymena*, respectively, the ciliary rows are no longer arranged in orderly parallel lines and are therefore mis-oriented relative to the overall body axis, but within each row the relative position of individual ultrastructural features associated with each cortical unit remain unaffected [46,47]. In this case, global positional cues apparently do not affect the local geometrical relation of the components of a cortical unit.

**Figure 3:**
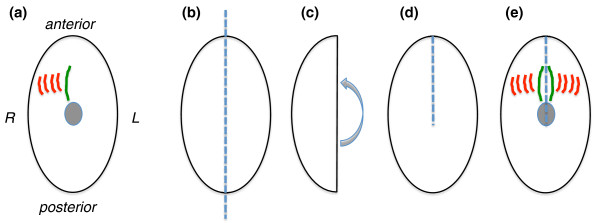
**Global versus local information in cell morphogenesis as revealed by grafting in *Stylonychia *(modified from diagram in **[46]). **(a) **Highly schematic view of paroral structures during normal development in *Stylonychia*, showing paroral membrane (PM; green) flanked by fronto-ventral-transverse (FVT) cirri (red). The oral primordium is shown as a grey disc. Other ciliary structures are not shown. Left and right (by convention given from the cell's perspective) are as indicated. **(b) **Grafting experiment of Grimes and L'Hernault [7]. **(c, d) **The cell was cut lengthwise, then the right half folded over (c), thus placing the former posterior region to the left of the former anterior region (d) with the join shown as a dotted line. **(e) **When PM and FVT cirri form, two sets are formed, one on the left and one on the right. The set on the left has inverted chirality because the structures have an anterior-posterior order consistent with the overall cell body axis after grafting, while the left-right ordering is consistent with the local position of these structures in the right half before cutting and folding.

The inheritance of inverted ciliary rows reported by Beisson and Sonneborn, by contrast, is a clear-cut example of local information, in the form of the relation between mother and daughter centrioles, giving rise to a stably propagating global structure (the inverted ciliary row). The most extreme interpretation of these experiments would be that local information is sufficient to explain cellular organization and that global information may have a much less critical role than had been previously imagined. This raises the question whether some structures respond purely to global information and others to purely local, or do all structures respond to both? A series of microsurgical experiments in the ciliate *Stylonychia *has helped clarify this issue [7]. This organism normally forms a series of ciliary rows called the paroral membranes (PMs) flanked by a another set of cilia-based structures called fronto-ventral-transverse (FVT) cirri, which work together to create a flow of fluid towards the mouth, allowing the cell to feed (Figure 3a). The chiral relation of the PMs relative to the FVT cirri (the FVT cirri are always on the right hand side of the PM when the cell is viewed from its ventral surface) raises the question of whether this arrangement reflects global left-right asymmetry of the whole cell, or local left-right asymmetry of the PM-FVT cirri relationship. To distinguish these possibilities, individual cells of *Stylonychia *were cut in half lengthwise and then the right half folded back on itself to join the former anterior and posterior ends together (Figure 3b-d). When the folded cell healed, the left half formed PMs and FVT cirri that retained the same left-right asymmetric arrangement that they would have had if they had still been on the right side - that is, rotated 180 degrees - but took on the anterior-posterior arrangement appropriate to the overall cell body axis, with the result that the structures were mirror images of those formed on the right half of the same cell [7] (Figure 3e). This shows that some aspects of the paroral array of cilia, such as the left-right arrangement of the structures, are dictated by the local relation between the cortical elements (basal bodies and associated structures) while other aspects, such as the anterior-posterior arrangement of the structures, are imposed by a global cell polarity cue.

The distinction between global and local information is nicely illustrated by studies in the outer hair cells of the mammalian cochlea. These cells form orderly and highly directed chevron-shaped arrays of stereocilia (Figure 2a) whose orientation is dictated by planar cell polarity, a global clue expressed as asymmetry in one plane of a tissue [48] and at the level of the whole cell [49]. But interestingly, if planar cell polarity is abrogated by mutations, the stereocilia still form normal-looking chevrons with a clear orientation, but now the direction is random with respect to the other cells in the tissue [49]. These studies suggest that, just as seen in *Stylonychia*, local information specifies the detailed organization of a complex subcellular structure (in this case the orderly rows of stereocilia in a chevron pattern), while global information specifies the orientation and position of the structure within the cell itself (in this case relative to planar cell polarity cues).

Finally, it is worth mentioning briefly that local structure could feed back to affect global organization. For example, in vertebrate multiciliated epithelia such as those found in the airway or oviduct, arrays of oriented cilia drive directed fluid flows [50]. In these cells, the orientation of cilia responds to both fluid flow and planar cell polarity cues [51], with the cilia in turn generating a fluid flow-field that extends over the whole tissue to influence the orientation of other cilia [52,53], thus blurring the usual distinction between local and global levels of organization. Another example of the inter-relation between local and global information is seen in the green alga *Chlamydomonas*, where the mother centriole position is specified by global cell polarity cues, while daughter centriole position is specified by the mother, so that if daughter centrioles become detached from the mother centriole, they wind up occupying random positions in the cell [54].

## Scaling

So far we have focused on the position and orientation of cellular structures. An equally fundamental problem in cell geometry is how the sizes of the different organelles in a cell are controlled so as to be appropriate relative to the overall size of the cell. Experimental studies have shown that a number of cellular structures scale with overall cell size, including nuclei [55], contractile rings [56], mitochondria [57], and mitotic spindles [58]. Although the problem of scaling of cellular structures to the size of the cell was posed over 100 years ago by Thomas Hunt Morgan in the context of experiments in *Stentor *[59], the mechanisms that couple cell size to organelle size remain largely a mystery [60]. Indeed, very little information currently exists about the mechanisms that control the size of organelles, although studies in organelles such as cilia and flagella, which have simple geometries, have some promise. For the case of flagella in the green alga *Chlamydomonas*, it appears that length is determined by the steady-state balance between continuous length-independent disassembly of the flagellar microtubules and continuous assembly of new tubulin onto the distal tips, a process that is inherently length-dependent [61]. Such a model suggests that the key to organelle size regulation is the balance of assembly and disassembly, and if either the assembly or disassembly rate is inherently size-dependent, then it is possible for a very simple steady-state model to explain size determination. But to further link such a model to the scaling of structures relative to the overall cell size, one must understand how either the assembly or disassembly rate is linked to cell volume, and this is so far unclear in most systems.

Spindles provide a particularly important instance of scaling because their length has to be related precisely to cell diameter in order to ensure proper division into the two daughter cells. Mitotic spindle length scaling has been investigated in cleavage divisions of *Xenopus*, which have the convenient property that cell size decreases by a factor of two at each division. In this system, spindle length has been found to be an increasing function of cell size, but only over a limited size range [58] - in sufficiently large cells, spindle length becomes independent of the size of the cell.

Cell size is not the only determinant of spindle length, however. Because spindle length is determined by the interplay of numerous molecular players, including multiple different motor proteins [62], it should also vary as a function of cytoplasmic protein composition, as illustrated by recent experiments comparing spindle lengths in different *Xenopus *species. *Xenopus tropicalis*, a small relative of *Xenopus laevis*, forms correspondingly smaller meiotic spindles, suggesting that meiotic spindle length is subject to length scaling just as mitotic spindle length is. Experiments in which egg extracts from *X. laevis *or *X. tropicalis *were added to sperm nuclei from *X. laevis *sperm showed that the *X. tropicalis *extracts produced substantially shorter spindles than the *X. laevis *extracts [63]. Although this was interpreted as reflecting a molecular basis for spindle length scaling, it is apparently a very different type of scaling because it is not an intrinsic property of the spindle assembly mechanism that responds to cell size, since the experiments were done in large volumes *in vitro*.

We therefore need to draw a distinction between two fundamentally different notions of scaling, which we will call direct scaling and programmed scaling. Direct scaling, which corresponds to the standard use of the term 'scaling' in physics, means a situation in which the size of a structure varies as a function of the size of the cell because the process that builds the structure is directly sensitive to the size of the cell, so that if the cell size were altered by some artificial means the spindle would rescale accordingly. Programmed scaling, by contrast, would correspond to situations in which the size of a structure is controlled by expression levels or enzymatic activities that are not themselves sensitive to the size of the cell, but which may have been tuned by evolution to yield a structure of a size appropriate for the typical cell size in that organism. Programmed scaling is thus an entirely different concept from that usually expressed by the term 'scaling' in physics and biology. Considering the above, we would say that the cell size-driven mitotic scaling during cleavage divisions of *Xenopus *[58] would fall into the class of direct scaling, while the extract composition-driven meiotic spindle length variation between different *Xenopus *species [63] would fall into the class of programmed scaling.

## Where do we go from here?

We can identify two clear-cut needs that, if addressed, would put us on a stronger footing for future understanding of the origins of cell geometry. First, we note that at present we have no rigorous way to define the level of organization in a cell. We are thus left to our subjective visual impression to say that cell type X is more organized than cell type Y. Often in science, major progress follows once a previously subjective concept is given a rigorous quantitative definition. For cellular complexity, we currently lack a good way to quantify organization and polarization that would allow us, for example, to determine if a particular perturbation, such as a mutation or drug treatment, resulted in a statistically significant decrease in organization. In the absence of a numerical measure of organization, concepts like statistical significance cannot be applied. While one might at first think that trying to represent the complexity of a cell with a single number could be a fruitless enterprise, one need only look to the concept of entropy, a single number that can be used to define the degree of order in a wide range of different physical systems, to see how useful such a simplified measure can be. However, entropy is probably not the appropriate metric for organization in cells since it can be more strongly influenced by small-scale positions of molecules rather than large scale spatial structures. We require a measure of complexity appropriate to the scale of organelles and subcellular structures. Recent developments in methods to quantify cellular organization in statistical terms [64] provide one possible way to build numerical descriptors of order, by providing numerical values for a set of shape description features for each cell image. This then would allow one to test whether a particular cell type shows a greatly restricted range of structural features compared to the total range of values that such descriptors could take. Such a restriction in shape feature values would be an indicator of order in cell structure.

A second key need is efforts to develop interesting structurally complex cell types into tractable model systems, particularly cell types in which complex structures arise in a cell-autonomous manner. For unicellular organisms such as *Stentor *this would mean sequencing their genomes and developing methods for reverse genetics, such as RNA interference. Such work is currently in progress in many labs, including that of the author. For vertebrates, this means greater effort in culturing cell types of structural interest - for example, cochlear hair cells - or else developing more approaches to studying their development *in situ *using *in vivo *imaging [65].

Probably the most important thing we can do, at this point, is simply keep the question in focus. Every time we see a cell with an interesting structure, there is a question to be asked concerning how that structure arises, and the answers to such questions are likely to be a treasure trove of new insights into the molecular biology of the cell.
